# Reconstructive osteotomy of fibular malunion: review of the literature

**DOI:** 10.1007/s11751-011-0107-2

**Published:** 2011-04-06

**Authors:** Remco J. A. van Wensen, Michel P. J. van den Bekerom, René K. Marti, Ronald J. van Heerwaarden

**Affiliations:** 1 Department of Orthopaedic Surgery, Sint Maartenskliniek Woerden, P.O. Box 8000, 3440 JD Woerden, The Netherlands; 2 Department of Orthopaedic Surgery, Academic Medical Centre, Amsterdam, The Netherlands; 3 Academic Medical Centre, Amsterdam, The Netherlands; 4 Klinik Gut, St. Moritz, Switzerland; 5 Limb Deformity Reconstruction Unit, Department of Orthopaedic Surgery, Sint Maartenskliniek, Woerden, The Netherlands

**Keywords:** Ankle, Fracture, Osteotomy, Malunion, Operative procedures, Fibula

## Abstract

The treatment of ankle fractures has a primary goal of restoring the full function of the injured extremity. Malunion of the fibula is the most common and most difficult ankle malunion to reconstruct. The most frequent malunions of the fibula are shortening and malrotation resulting in widening of the ankle mortise and talar instability, which may lead to posttraumatic osteoarthritis. The objective of this article is to review the literature concerning the results of osteotomies for correcting fibular malunions and to formulate recommendations for clinical practice. Based on available literature, corrective osteotomies for fibular malunion have good or excellent results in more than 75% of the patients. Reconstructive fibular osteotomy has been recommended to avoid or postpone sequela of posttraumatic degeneration, an ankle arthrodesis or supramalleolar osteotomy. The development of degenerative changes is not fully predictable; therefore, it is advisable to reconstruct a fibular malunion soon after the diagnosis is made and in presence of a good ankle function. Recommendations were made for future research because of the low level of evidence of available literature on reconstructive osteotomies of fibular malunions.

## Introduction

The treatment of ankle fractures has a primary goal of restoring the full function of the injured extremity. Restoring anatomical alignment, with a fully congruent mortise, and restoring joint stability are the keys to a successful treatment, conservatively or by open reduction and stabilization [1, 2].

In ankle fractures, the talus may be displaced. Ramsey et al. found in a classic biomechanical model that one millimetre lateral displacement of the talus causes a 42% reduction in the tibiotalar contact area [3]. Other cadaveric studies confirmed these biomechanical changes which may lead to arthritis of the ankle joint and a poor functional outcome [4, 5]. Therefore, the distal fibula plays a main role in the anatomical reduction of displaced ankle fractures, restoring ankle stability and congruity of the ankle mortise [6, 7]. Malunion of the fibula is the most common and most difficult ankle malunion to reconstruct [8]. The most frequent malunions of the fibula are shortening and malrotation resulting in widening of the ankle mortise and talar instability, which may lead to posttraumatic osteoarthritis [7–10].

In 1936, Speed and colleagues were the first who described a fibular osteotomy as a possible treatment for malunited ankle fractures [11]. In the last three decades, many other case series followed describing the results of these corrective osteotomies. The objective of this article is to review the literature concerning the results of these osteotomies for correcting fibular malunions and to formulate recommendations for clinical practice.

## Materials and methods

The literature search was limited to published original studies including adult male and/or female patients with a diagnosis of a distal fibular malunion corrected by lengthening and/or derotation osteotomy. The main databases Pubmed/Medline, Cochrane Database of Systematic Reviews, Cochrane Clinical Trial Register, Database of Abstracts on Reviews and Effectiveness, Current Controlled Trials, National Research Register and Embase were searched from 1960 to October 2007 to identify studies relating to the results of using osteotomy for malunited ankle fractures. From the title and the abstract, two reviewers (RW, MB) independently reviewed literature searches to identify potential relevant studies for full review. From the full text, these reviewers selected the studies for inclusion. Disagreement was resolved by consensus or by third party adjudication (RH). The applied search terms were: ‘ankle injury’, ‘fractures’, ‘lengthening’, ‘derotation’, ‘malunion’ and ‘osteotomy’. The result was combined with an electronically backward search and checked manually for related articles. Furthermore, the lists of references of retrieved publications were manually checked for additional studies potentially meeting the inclusion criteria and not found by the electronic search. Case reports were included. The search was restricted to articles written in the English, German and Dutch language.

Methodological quality of the included studies was assessed by two reviewers (RW, MB) according the Level of Evidence scoring system. (Table 1) (http://www.cebm.net/) Any disagreement was resolved by consensus. Grades of recommendation of the various treatment options were formulated based on level of evidence supporting that treatment. Data were independently extracted by two reviewers (RW, MB) Results of different types of osteotomy were separately analysed.

**Table 1:** Level of evidence and grades of recommendation

Level of evidence
Level I: high quality prospective randomized clinical trial
Level II: prospective comparative study
Level III: retrospective case–control study
Level IV: case series
Level V: expert opinion and case reports
Grades of recommendation
Grade A treatment options are supported by strong evidence (consistent with Level I or II studies)
Grade B treatment options are supported by fair evidence (consistent with Level III or IV studies)
Grade C treatment options are supported by either conflicting or poor quality evidence (Level IV studies)
Grade D when insufficient evidence exists to make a recommendation

It was the initial intention of the authors to use a strict methodology for paper selection, focusing on objectively measurable variables, separate evaluation of different fracture types and different associated injuries, and randomized controlled trails. These scientific standards had to be abandoned, however, as almost none of the available papers fulfilled the above-mentioned criteria.

## Results

Seventeen articles were found and met our inclusion criteria. Two important articles were excluded because of preventing selection bias. One article contained patient data published in an other journal, and the other article contained a case series of 6 patients which had also been published previously in a case series of eight patients [12, 13]. The remaining fifteen studies that were included contained level of evidence IV and V results [7, 14–27]. Table 2 shows all included studies and available patient data. The number of patients, time to revision, follow-up period, type of osteotomy, use of syndesmotic fixation, use of bone grafts and finally the complications and postoperative weight-bearing advice were mentioned.

**Table 2:** Included studies and available patient data

Study	Year	Country	Patients	Level of evidence	Time from initial trauma (m)	Follow-up (m)	Osteotomy (T/O/Z)	Osteotomy (L/R)	Syndesmotic fixation	Bone graft	Cast	Partial weight-bearing after…	Full weight-bearing after…	Nonunion	Deep infection
Hughes [14]	1976	USA	28	IV	nm	nm	nm	L	nm	nm	nm	nm	nm	nm	nm
Offierski et al. [15]	1982	Canada	11	IV	8 (3–36)	40 (12–84)	Varied	L/R	Yes	Yes	nm	nm	nm	nm	nm
Fogel et al. [16]^b^	1982	USA	5	IV	6 (1–16)	12 (4–18)	nm	L/R	Varied	Varied	nm	nm	nm	nm	nm
Weber BG et al. [12]	1985	Switzerland	23	IV	3–48	134 (60–144)	T	L/R	No	Yes	8w	Walking plaster	nm	0	0
Dehne et al. [18]	1986	USA	1	V	6	18	O	L/R	nm	Yes	nm	nm	nm	No	No
Austin [19]	1987	England	1	V	300	12	T	R	nm	nm	8w	nm	nm	No	No
Yablon et al. [20]	1989	USA	26	IV	72 (12–252)	84 (6–132)	T	L/R	Yes	Yes^a^	4w	4w	>4w with orthosis	1	1
Ward et al. [21]	1990	England	6	IV	12 (1–52)	18 (12–24)	T	L/R	Yes^a^	Yes	4–8w^c^	nm	nm	0	0
Marti et al. [7]	1990	Netherlands	31	IV	22 (>3)	60 (18–120)	T	L/R	Yes^a^	Yes	6w	nm	nm	0	1
Roberts et al. [22]	1992	USA	3	IV	3 (1–6)	33 (12–54)	Varied	L	Yes^a^	Yes	nm	nm	nm	0	0
Davis et al. [23]	1995	USA	3	IV	24 (no range)	34 (18–48)	Varied	L/R	Yes^a^	Yes^a^	4–5w	Varied	7–8w	0	0
Weber D et al. [24]	2001	Switzerland	8	IV	33 (5–60)	47 (18–102)	Varied	L/R	Yes	Yes^a^	6w	7–12w	12w	0	1
Weber M et al. [25]^b^	2003	Switzerland	3	IV	9 (3–13)	59 (46–80)	Varied	L/R	Varied	Varied	6w	7–12w	12w	0	0
Chao et al. [26]	2004	Taiwan	12	IV	28 (6–48)	34 (27–48)	O	L/R	Yes	No	6w	7–12w	12w	0	0
Eberl et al. [27]	2006	Germany	16	IV	10 (4–33)	44 (8–77)	O	L/R	No	Yes	1w	nm	6w	0	0

*L/R* lengthening and/or rotational, *nm* not mentioned, *T* transverse osteotomy, *O* oblique osteotomy, *Z* Z-osteotomy

^a^If necessary

^b^Only patients with fibular osteotomy were included

^c^Except 1 case

Table 3 presents the clinical results of the 177 included patients. One hundred and thirty-seven patients (77%) had a good or excellent result after osteotomy. There is a wide variation in the outcome measurements used and often no validated measurements were used.

**Table 3:** Clinical results

Study	Year	Patients	Main outcome measure	Excellent/Good	Fair/Poor
Hughes [14]	1976	28	nm	22	6
Offierski et al. [15]	1982	11	Burwell and Charnley	8	3
Fogel et al. [16]	1982	5	Joy, Patzakis and Harvey	1	4
Weber BG et al. [12]	1985	23	nm	17	6
Dehne et al. [18]	1986	1	nm	1	
Austin [19]	1987	1	nm	1	
Yablon et al. [20]	1989	26	nm	20	6
Ward et al. [21]	1990	6	Joy, Patzakis and Harvey	5	1
Marti et al. [7]	1990	31	Modified Weber rating scale	22	9
Roberts et al. [22]	1992	3	nm	2	1
Davis et al. [23]	1995	3	nm	2	1
Weber D et al. [24]	2001	8	nm	6	2
Weber M et al. [25]	2003	3	nm	3	
Chao et al. [26]	2004	12	Ankle Hindfoot Scale	11	1
Eberl et al. [27]	2006	16	Olerund and Molander	16	
Total		177		137	40
				77,40%	22,60%

Table 4 shows the objective measurements on the radiographic images used in the included studies. These measurements consisted of the talar tilting, talocrural angle, bimalleolar angle, ankle mortise geometry and progression of osteoarthritis. These measurements were not always mentioned, and in only 6 studies validated, objective measures scales were used.

**Table 4:** Objective radiological measurements

Study	Year	Talar tilt	Talocrural angle measure	Bimalleolar angle measure	Talar shift/ankle mortise	(Progression of) osteoarthritis	Measurement
Hughes [14]	1976	nm	nm	nm	nm	nm	nm
Offierski et al. [15]	1982	Yes	No	No	Yes	Yes	Burnwell and Charnley
Fogel et al. [16]	1982	No	No	No	Yes	No	Joy et al.
Weber BG et al. [12]	1985	No	No	No	Yes	Yes	nm
Dehne et al. [18]	1986	No	Yes	No	nm	Yes	nm
Austin [19]	1987	No	No	No	Yes	No	nm
Yablon et al. [20]	1989	No	No	No	Yes	Yes	No
Ward et al. [21]	1990	No	No	No	Yes	Yes	Joy et al. and Magnusson
Marti et al. [7]	1990	No	No	No	No	Yes	Modified Weber rating scale
Roberts et al. [22]	1992	No	No	Yes	Yes	Yes	nm
Davis et al. [23]	1995	Yes	Yes	Yes	Yes	No	nm
Weber D et al. [24]	2001	No	No	No	No	Yes	nm
Weber M et al. [25]	2003	No	No	No	Yes	Yes	nm
Chao et al. [26]	2004	Yes	Yes	No	Yes	Yes	nm
Eberl et al. [27]	2006	No	No	No	No	Yes	Magnusson

*nm* Not Mentioned

Authors’ conclusions for factors affecting clinical outcome after osteotomy for fibular malunions are shown in Table 5. These conclusions were based on their own results or on conclusions made in literature they support or referred to. Quality of reduction and osteoarthritis at the time of osteotomy were thought to be main factors affecting clinical outcome.

**Table 5:** Factors affecting clinical outcome

Study	Year	Age	Sex	Type of fracture	Initial treatment	Time to revision	Quality of reduction	Osteoarthritis at time of revision	Severity of malunion	Integrity of distal tibiofibular syndesmosis
Hughes [14]	1976	−	nm	nm	−	−	nm	nm	nm	nm
Offierski et al. [15]	1982	−	−	−	−	+	+	+	nm	nm
Fogel et al. [16]	1982	nm	nm	nm	nm	+	+	+	nm	nm
Weber BG et al. [12]	1985	nm	nm	nm	nm	−	+	+	−	nm
Dehne et al. [18]	1986	nr	nr	nr	nr	nr	nr	nr	nr	nm
Austin [19]	1987	nr	nr	nr	nr	nr	nr	nr	nr	nm
Yablon et al. [20]	1989	nm	nm	nm	nm	−	+	+	−	nm
Ward et al. [21]	1990	nm	nm	nm	nm	nm	+	nm	nm	nm
Marti et al. [7]	1990	−	nm	nm	nm	−	nm	−	−	nm
Roberts et al. [22]	1992	nm	nm	nm	nm	nm	+	nm	nm	nm
Davis et al. [23]	1995	nm	nm	nm	nm	nm	+	nm	nm	+
Weber D et al. [24]	2001	−	nm	nm	−	−	nm	+	nm	nm
Weber M et al. [25]	2003	nm	nm	nm	nm	nm	nm	nm	nm	nm
Chao et al. [26]	2004	nm	nm	nm	nm	−	+	+	nm	nm
Eberl et al. [27]	2006	−	nm	−	−	−	nm	+	nm	nm

Age, initial treatment and time to revision were referred by Weber BG

*nm* not mentioned, *nr* not relevant, *n* = 1

+ Affecting clinical outcome

− Not affecting clinical outcome

## Discussion

This article reviews the literature concerning osteotomies for fibular malunion. Most patients with malunited fractures of the ankle joint complain about pain, swelling and stiffness of the ankle joint, difficulty in walking and impairment of activities [9, 20, 23]. Secondary lateral rotation and abduction (lateral tilt) of the talus leads to a posttraumatic flat foot, followed by arthritic changes and contractures [7, 9, 12].

**Fig. 1:**
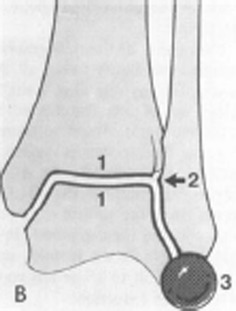
Three characteristics of the ankle on the 20° internally rotated anteroposterior view

The radiological diagnosis can be achieved on the 20° internally rotated anteroposterior view of the ankle. Three characteristic radiological abnormalities have been described as follows: 1, a joint space of which the line of the tibial plafond and the line of the surface of the talar dome are no longer strictly parallel, particularly on the medial side due to talar shift; 2, a broken ‘Shenton’s line of the ankle’; 3, a broken curve between the lateral part of the talar articular surface and the fibular recess (Fig. 1) [9, 12, 17, 23, 27–29].

Other measurements of importance to diagnose, preoperative planning and postoperative evaluation are the talar tilt, the talocrural angle and the bimalleolar angle. The talar tilt represents the angle between the line of the tibial plafond and the line of surface of the talar dome. In a normal ankle joint, these lines should be parallel [8, 15, 23, 28, 29]. The talocrural angle describes the angle between the line of the tibial plafond and the line through the tips of the malleoli. If the difference of this angle is ≥3° between the injured and the contralateral ankle, a fibular shortening is present [8, 23, 29]. The bimalleolar angle is described by the line connecting the malleolar tips and a vertical line following the fibular intramedular space, immediately superior of the ankle joint. Difference of 2.5° or greater between both sides suggests fibular shortening [23, 30]. Roberts et al. showed that the change of the bimalleolar angle with 1° represents 1 mm fibular shortening or lengthening after corrective osteotomy [22, 23].

Bilateral CT scan is the available method to confirm the incongruence of the lateral malleolus in the incisura fibularis tibiae [9, 13]. The radius of the distal fibula increases distally of the incisura fibularis tibiae, leading to lateralization of the external malleolus and widening of the ankle mortise [9]. Fibular malrotation is difficult to visualize on plain radiographs. If rotational malalignment is suspected, a CT scan with three-dimensional reconstruction should be considered [2, 8, 20, 23, 27, 28]. MRI examination is not necessary, but it can detect interposition of soft tissue, which may also be detected perioperatively. MRI has the added benefit of articular cartilage assessment [8, 9, 28]. Secondary reconstruction is indicated in the presence of reasonable ankle function and even in the presence of arthritic changes [7, 9]. The optimal time to perform reconstruction has not been defined clearly. Arthritic changes may be severe already after several months or may be minimal even after 2 or 3 years [12, 17, 26]. The period between the initial trauma and correction is not affecting clinical outcome, but correcting the malunion soon after the diagnosis and before osteoarthritis has developed is recommended. (Table 5) [20, 26, 28] The goal of any reconstructive intervention for malunion is to restore the anatomical alignment, joint congruency and joint stability of the ankle. Reconstruction may reduce the progression of degenerative changes and can decrease the symptoms of arthritis by decreasing instability and load on the arthritic locations of the joint [8, 12, 17, 28]. In most cases, reconstructive surgery consists of lengthening and derotation of the distal fibula. Three types of osteotomies have been described. Oblique or Z-osteotomies of the fibula are advised for correction of shortening and external rotation less than 10°. An oblique osteotomy through the old fracture is only indicated for a Weber B fracture and allows better correction but is difficult to perform. Transverse osteotomy is indicated for malunion after Weber C fracture and is always performed above the syndesmosis [8]. If external rotation is exceeding 10°, a transverse osteotomy allows an easier derotation and another benefit of the transverse osteotomy is the significant amount of lengthening that can be achieved [12, 17, 28]. A laminar spreader, a pin clamp and an AO compression device can be used as distractors for fibular lengthening [7, 9, 12, 17, 28, 31]. The secret of a successful reconstruction is the anatomic positioning of the external malleolus in the incisura fibularis tibiae. Debridement of the syndesmotic scar tissue is absolutely necessary to be able to lengthen the fibula, otherwise the fibula cannot be pushed downwards to the tibiofibular joint. After debridement, if there are still remaining fibres of the syndesmosis, the ankle may be stable, otherwise a syndesmotic position screw has to be placed, e.g., through the plate used to fix the osteotomy. If syndesmotic injury is suspected on direct visualization during surgery or by C-arm stress views, syndesmotic fixation by noncannulated fully threaded cortical screws through four cortices is recommended [8, 9, 20]. Bone grafts are advised to fill an osteotomy gap of more than 3 mm [20]. Choices of bone grafts vary between structural allografts or iliac crest structural autografts or cancellous bone autografts [32]. Cancellous graft out of the supramalleolar area is most often sufficient.

Postoperatively, patients should be placed in a nonweight-bearing removable cast for 2 weeks allowing patients to train their ankle function. According to most authors the patients should be placed in a below-the-knee-nonweight-bearing cast for at least 6 weeks. Then the patiënt starts with partial weight-bearing for another 6 weeks followed by full weight-bearing. Most patients will achieve a good ankle function after this semi-functional treatment [8, 9].

In our review, overall subjective outcomes after fibular osteotomy are good or excellent in more than 75% of the patients. These good to excellent results were already described by Offierski et al. and Fogel et al. [15, 16] Standardized subjective and radiological measures were not always used and if used, there was a broad variation of measures that were therefore not comparable. Clinical factors affecting outcome were mostly thought to be the quality of reduction and the presence of osteoarthritis at the time of revision. However, Marti and colleagues suggested that the clinical outcome was mainly related to the preoperative mobility of the ankle joint. Only a severely disturbed ankle function was considered a contraindication for reconstructive surgery [7].

According to several authors, the onset and/or progression of osteoarthritis in malunited ankles is reduced after performing a corrective osteotomy. Only advanced degenerative changes were considered as a contraindication for an osteotomy of the distal fibula by most authors [8, 12, 15, 17, 20, 23, 28, 29]. Unfortunately, postoperative degenerative changes occur. Initially, these patiënts have to be treated with anti-inflammatory medications and braces. In severe arthritic changes, a custom ankle foot orthosis may be indicated. Surgical alternatives, including ankle replacement, ankle fusion and cheilectomy, are available after failure of all nonoperative treatments [8].

Limitations of this review are the low number of patients per study, the lack of evidence and diversity of outcome measurements that made pooling of the results not realistic. There is low level of evidence of the included studies resulting in only a grade C (Table 1) level of recommendation of different treatment options. Therefore, only preliminary conclusions can be drawn and some suggestions for further research can be made. However, the question remains if research of a higher level of evidence is achievable. In future studies, the use of well defined and validated functional outcome measures is preferable. The use of standardized outcome measurements is encouraged to facilitate meta-analyses and between trial comparisons. Larger and well-documented case series are needed to reveal the factors that influence the outcome of corrective osteotomies for fibular malunions.

In summary, reconstructive osteotomy for fibular malunion is well tolerated. Based on available literature, a corrective osteotomy for fibular malunion has a good or excellent result in more than 75% of the patients. Reconstructive fibular osteotomy has been recommended to avoid or postpone an ankle arthrodesis or supramalleolar osteotomy. The development of degenerative changes is not fully predictable; therefore, it is advisable to reconstruct fibular malunion soon after diagnosis is made and in the presence of a good ankle function [7]. Future research should focus on the long-term outcome, the predictors of a good outcome and the comparison with nonoperative treatment. These studies should use uniform and patient-based outcome measures resulting in higher level of evidence research results.
